# Progress of Induced Pluripotent Stem Cell Technologies to Understand Genetic Epilepsy

**DOI:** 10.3390/ijms21020482

**Published:** 2020-01-12

**Authors:** Bruno Sterlini, Floriana Fruscione, Simona Baldassari, Fabio Benfenati, Federico Zara, Anna Corradi

**Affiliations:** 1 Department of Experimental Medicine, University of Genova, Viale Benedetto XV, 3, 16132 Genoa, Italy; bruno.sterlini@unige.it; 2 Center for Synaptic Neuroscience and Technology, Istituto Italiano di Tecnologia, Largo Rosanna Benzi 10, 16132 Genoa, Italy; fabio.benfenati@iit.it; 3 Department of Neurosciences, Rehabilitation, Ophthalmology, Genetics, Maternal and Child Health, University of Genoa, Largo P. Daneo 3, 16132 Genoa, Italy; Floriana.Fruscione@unige.it; 4 Unità Operativa Complessa Genetica Medica, Istituto di Ricovero e Cura a Carattere Scientifico Giannina Gaslini, Genova Italy, Via G. Gaslini 5, 16147 Genoa, Italy; simonabaldassari@gmail.com; 5 Istituto di Ricovero e Cura a Carattere Scientifico, Ospedale Policlinico San Martino, Largo Rosanna Benzi 10, 16132 Genoa, Italy

**Keywords:** epilepsy, induced pluripotent stem cells, disease modeling, cerebral organoid, neuronal excitability, transplantation

## Abstract

The study of the pathomechanisms by which gene mutations lead to neurological diseases has benefit from several cellular and animal models. Recently, induced Pluripotent Stem Cell (iPSC) technologies have made possible the access to human neurons to study nervous system disease-related mechanisms, and are at the forefront of the research into neurological diseases. In this review, we will focalize upon genetic epilepsy, and summarize the most recent studies in which iPSC-based technologies were used to gain insight on the molecular bases of epilepsies. Moreover, we discuss the latest advancements in epilepsy cell modeling. At the two dimensional (2D) level, single-cell models of iPSC-derived neurons lead to a mature neuronal phenotype, and now allow a reliable investigation of synaptic transmission and plasticity. In addition, functional characterization of cerebral organoids enlightens neuronal network dynamics in a three-dimensional (3D) structure. Finally, we discuss the use of iPSCs as the cutting-edge technology for cell therapy in epilepsy.

## 1. Introduction

The understanding of the mechanisms of neuronal function is of great importance to acquire an insight into the physiopathology of neurological diseases. The knowledge about neuronal phenotypes in human diseases has been hampered by the very limited access to Central Nervous System (CNS) samples from patients. In this context, the possibility of generating human-induced Pluripotent Stem Cells (iPSCs) by the genetic reprogramming of differentiated human cells represents a major technological advance to study the pathogenesis of neurological disorders [1]. Since that time, several groups devoted large efforts in the attempt to differentiate the iPSCs towards diverse cell types, such as neurons, muscle cells or cardiomyocytes [2].

Human iPSC-derived neurons were used to model the different types of neurological diseases from developmental to neuropsychiatric and neurodegenerative diseases [3,4,5]. Moreover, these cells can be used also to test the efficacy and toxicity of new and old drugs, and have the great potential to be used in cell therapy and personalized medicine [6].

iPSC-derived neurons represent a unique model to study neurological diseases of genetic origin, as they preserve the patient’s genetic background, which influences and affects disease onset and progression.

In addition, genome editing approaches, such as the transcriptor activator-like effector nuclease (TALEN) or the clustered regularly interspaced short palindromic repeats/protein 9 nuclease (CRISPR/CAS9) have made it possible to investigate the physiopathological role of specific mutations by generating patient-derived isogenic iPSC lines [7].

Epilepsy is a very common condition, affecting up to 1% of the population, and is characterized by spontaneous, recurrent and self-limiting epileptic seizures. Despite the availability of many anti-epileptic drugs, approximately 30–40% of patients have medically refractory seizures. Epilepsy includes different forms according to the semiology of seizures, the age of onset, the presence of associated symptoms and the outcome. In focal epilepsies, seizures start in a limited area of the brain. In generalized epilepsies, seizures begin simultaneously in both hemispheres. The epilepsies are also grouped based upon the etiology, being acquired or genetic, with either identified or presumptive genetic etiology [8].

With the advent of next-generation sequencing, over 140 genetic loci associated with various forms of epilepsies were identified in the last few years. Epilepsy-associated genes encode for ion channel and synaptic proteins, as well as cell adhesion molecules, signaling proteins and transcription factors. The mechanisms through which mutations in such different genes lead to epileptic seizures are largely unknown, although they ultimately lead to brain hyperexcitability [9].

Brain function results from the integration of multiple neuronal circuits, with high levels of regulation and plasticity. Understanding how genetic mutations affecting diverse molecular pathways may lead to the unbalance of neuronal networks is critical in identifying innovative therapeutic approaches. In this review, we examine the most recent papers that used iPSC-derived neurons to study genetic epilepsy, with particular focus on the specific forms listed in Table 1, and then discuss the developments in iPSC technologies that may significantly advance experimental epileptology.

## 2. iPSC-Based Human Models for Epilepsy

### 2.1. Epileptic Encephalopaties

#### 2.1.1. Dravet Syndrome (EIEE6)—SCN1A

Dravet syndrome (DS) is a severe, infantile-onset epilepsy syndrome associated with cognitive deficit and developmental delay in language, motor function, learning and social skills [10]. Seizures are refractory to all currently available treatments. Most DS patients carry de novo heterozygous mutations in the SCN1A gene, encoding the α-subunit of the voltage-gated Na^+^ channel 1.1 (Nav 1.1). SCN1A mutations result in an impaired Na^+^ channels function and decreased neuronal excitability [11,12].

Studies in mouse models showed that the Nav1.1 subunit is more expressed in GABAergic inhibitory neurons, and decreased excitability in these neurons causes an excitation/inhibition unbalance, triggering epileptic seizures. Interestingly, among the diverse subclasses of interneurons, cortical and hippocampal parvalbumin and somatostatin interneurons display a higher expression of Nav1.1, and are therefore more affected [13,14,15,16,17]. Other studies in mouse models produced conflicting results. Favero et al. showed the presence of parvalbumin interneurons hypoexcitability only for a limited time window in young mice during development, suggesting that the chronic epilepsy of DS patients is not attributable to this class of neurons, that normalize their activity later in adulthood [18]. Moreover, mutations in Nav 1.1 channels have different effects depending on the mouse genetic background [19]. Thus, investigating the effects of DS mutations in a human genetic context emerged as a primary goal.

Starting from 2013, few groups generated iPSCs from the fibroblasts of DS patients with different mutations. Parent and collaborators differentiated iPSCs into a mixed culture of excitatory and inhibitory forebrain neurons, and detected higher Na^+^ currents and hyperexcitability in both neuronal subtypes, an opposite phenotype compared to the reported mouse models [20]. A second study by Jiao et al. reported similar results [21]. In contrast, other papers [22,23,24,25] reported lower Na^+^ currents and hypoexcitability in iPSC-derived GABAergic interneurons carrying SCN1A mutation, in agreement with mouse models, whereas less information was reported for excitatory neurons. Phenotypic variability in studies with human cells may lie in differentiation protocols, generating inhibitory neurons at different degrees of maturation. However, in these studies, iPSC-derived inhibitory neurons were mostly identified by GABA or GAD67 positivity, and any quantification of specific neuronal subclasses is lacking.

To clarify this point, Sun et al. differentiated iPSCs from the fibroblasts of a pair of twins, carrying the Nav1.1 mutation, into dorsal telencephalon excitatory neurons and ventral telencephalon Medial Ganglionic Eminence-derived interneurons [26]. In agreement with the mouse model, the excitatory neurons displayed no phenotype, whereas inhibitory neurons showed lower Na^+^ currents and hypoexcitability, that were rescued by a re-expression of wild type SCN1A. The analysis of iPSC-derived GABAergic neurons showed that most of them were positive for calretinin, and only a few for somatostatin. Parvalbumin-positive interneurons, the specific subclass of inhibitory interneurons affected in DS, could not be generated. Recently, new protocols of GABAergic differentiation, successful in producing a large number of parvalbumin interneurons, will allow the investigation of the human DS interneuron phenotype (see improvements in 2D cultures). Finally, DS-iPSC-derived GABAergic neurons were recently used to test the efficacy and therapeutic action of a recently developed drug Cannabiol, showing that these cells are a suitable model to test the drug mechanism of action and toxicity [27].

#### 2.1.2. Malignant Migrating Partial Seizures of Infancy (MMPSI)(EIEE14)—KCNT1

*KCNT1* mutations lead to migrating partial seizures of infancy (MMPSI), a severe and pharmacoresistant early onset epileptic encephalopathy [28].

KCNT1 is an Na^+^-activated K^+^ channel (called Slack) that plays a role during hyperpolarization and contributes to returning the membrane potential to the resting state after neuronal firing [29]. KCNT1 missense mutations cause a gain of function, and in heterologous systems, lead to an increased activity of the channel [30]. Unfortunately, the only animal model available is a KCNT1 Knockout (KO) mouse. In this model, Bausch et al. observed the impairment of both reverse learning memory and adaptation to new environments, but they did not observe seizures [31].

To further explore the physiopathology of this disorder, Quraishi et al. generated an iPSC line, carrying a homozygous P924L gain of function variant by gene-editing, and differentiated it into forebrain neurons [32]. They performed a complete electrophysiological characterization by single neuron and network recordings. First, they confirmed an increase of Na^+^-dependent K^+^ currents in iPSC-derived neurons, as shown in the heterologous system. In addition, they observed hyperexcitability both in single neurons by current clamp and in neuronal networks by Multiple Electrode Array (MEA) technology. Furthermore, MEA analysis suggested that the altered timing of the high-frequency firing in the networks of KCNT1-mutated neurons is responsible for the increased synchrony of neuronal clusters. Accordingly, they proposed to define the KCNT1 encephalopathy as a disorder of hypersynchrony. In the future, it will be interesting to compare these iPSC-derived neurons with those obtained from heterozygous patients, carrying their own genetic background.

#### 2.1.3. Female-Limited Epilepsy and Cognitive Impairment (EIEE9)—PCDH19

PCDH19 is an X-linked gene encoding for delta2-protocadherin, a cell adhesion molecule highly expressed during development. Loss of function mutations in PDCH19 lead to a childhood epileptic encephalopathy associated with a spectrum of neurological signs including epilepsy, intellectual disability and autistic traits [33,34]. This phenotype manifests selectively in heterozygous females, does not occur in males, and displays a unique “inverse” X-linked pattern of inheritance.

A cellular interference model, based on the concomitant presence of PCDH19-positive and -negative neural progenitors impairing the communications between cell populations, has been proposed. Pederick et al. showed an increased in vitro mobility of neurons from PCDH19 KO mouse without evidence of any gross impairment in brain development [35]. Furthermore, they demonstrated that PCDH19 mosaic expression in heterozygous mice is responsible for differential adhesion affinities in neural progenitors, leading to an abnormal allocation of these cells and their neuronal progeny, potentially explaining the molecular basis of this pathology [36]. However, the animal models did not fully recapitulate the severity and clinical features of the pathology [37].

Homan et al. developed iPSC-derived neurons from PDCH19 mutant patients, and found that a loss of function of PCDH19 cause a premature and increased neurogenesis of neural stem and progenitor cells [38]. Furthermore, Compagnucci et al. observed a PCDH19 protein localization at the lumen by studying neural rosettes obtained from the iPSCs of healthy subjects, suggesting a potential role in the apicobasal polarity of neuroprogenitors [39]. These data support an implication of PCDH19 in cell adhesion dynamics causing a different timing of neuronal development also in the human cell model.

### 2.2. Cortical Malformation and Epilepsy

#### 2.2.1. Tuberous Sclerosis—TSC1 and TSC2

Tuberous sclerosis complex (TSC) is an autosomal dominant multisystem genetic disorder characterized by benign tumors in multiple organs. Glioneuronal hamartomas (i.e., cortical tubers) are the most common CNS manifestations of TSC characterized by dysmorphic neurons, astrogliosis and giant cells. They affect around 85% of patients, and frequently result in refractory epilepsy, intellectual disability and autism spectrum disorder [40]. TSC is caused by loss of function mutations in either the TSC1 or TSC2 genes, that are the key inhibitory component of the mTOR pathway, a central regulator of cell development, growth and survival. TSC patients carry a germline heterozygous mutation in either TSC1 or TSC2, but the formation of cortical tubers appears to be variable, and with random positioning and dependence on the occurrence of “second-hit” mutations [41].

This model indicates that biallelic inactivation, due to a somatic mutation in the second TSC allele, occurs in a small population of neural progenitor cells responsible for altered differentiation, development and neuronal migration.

In the animal model, the complete deletion of TSC1/2 is embryonic lethal, and to overcome this issue, many groups developed conditional KO models, demonstrating alterations in neuronal differentiation, survival, migration, morphology, excitability and synaptic plasticity that resemble that which was observed in a human post mortem brain [42,43]. However, rodent models lack specific neural progenitor cells, and are unable to reproduce the complexity of the human cortex.

Multiple groups developed iPSC-derived neurons carrying monoallelic and biallelic TSC mutations. Biallelic mutant cells display transcriptome alterations, closely related to those observed for human cortical tubers, and show an active inflammatory state and increased metabolic activity. In addition, neuroprogenitors, neuronal and glial cells show somatic hypertrophy and neuronal delayed maturation [44,45]. Electrophysiological analyses have been undertaken to explore those mechanisms underlying hyperexcitability. In 2016, Costa et al. observed, by whole-patch clamp, a strong decrease of spontaneous miniature excitatory postsynaptic currents (mEPSCs), likely related to the morphological alterations of neurons [46]. On the contrary, Nadadhur et al. found higher spontaneous neuronal firing in the TSC neuronal network, measured by MEA, suggesting an increased neuronal activity [47]. More recently, Winden et al. confirmed the increased activity levels of human TSC2−/− neurons by MEA, showing also a difference between control and heterozygous networks. Hypersynchronous neuronal discharges were also found in both TSC2+/− and TSC2−/− neurons [48].

#### 2.2.2. Lissencephaly—TUBA1A

Numerous mutations in Tubulin isotype genes have been associated with epileptogenic human cortical malformations. Within this heterogeneous family, mutations of TUBA1A lead to lissencephaly (i.e., smooth and without gyri cerebral cortex), cerebellar hypoplasia, corpus callosum agenesis, severe developmental delay and epileptic seizures.

Keays et al. generated a mouse model carrying the p.S140G TUBA1A mutation and observed altered neuronal migration responsible for abnormal architecture in the cortex and hippocampus [49]. More recently, Belvindrah et al. showed defects of neuronal migration (such as lower speed, loss of directionality and excessive branching) along the rostral migratory stream. In particular, they observed an impaired nucleus–centrosome (N–C) coupling essential for neuronal saltatory migration [50]. However, as observed for other brain disorders, modeling lissencephaly in animal models is limited by the physiological absence of cortical gyri.

To explore the TUBA1A function in a human context, Bamba et al. developed iPSC-derived neuroprogenitors from lissencephalic patients carrying TUBA1A mutation (p.N392S) using the neurosphere technology. They found a reduced neurite extension from the neurosphere which may represent a critical aspect of lissencephaly pathogenesis [51]. Further developments of iPSC technology toward cerebral organoids will provide suitable models to recapitulate and study human neuronal migration.

### 2.3. Idiopathic Epilepsies

#### PRRT2

Mutations in the proline-rich transmembrane protein 2 (PRRT2) gene cause a wide spectrum of neurological diseases, ranging from benign familial infantile epilepsy to paroxysmal kinesigenic dyskinesia and migraine. Most of the patients carry the same frameshift mutation that generates a premature stop codon and a truncated protein leading to a functional haploinsufficiency.

PRRT2 is a neuronal protein expressed on the plasma-membrane and at synaptic level, where it plays a role during Calcium-dependent neurotransmitter release [52,53]. The constitutive PRRT2 KO mouse recapitulated many of the phenotypic traits of human PRRT2-linked disorders, including paroxysmal dyskinesias and higher seizure propensity [54].

Fruscione et al. generated iPSCs from three siblings of a unique consanguineous family in which both parents carry heterozygous PRRT2 mutations, and therefore two of them are homozygous and show a more severe paroxysmal phenotype associated with intellectual disabilities [55]. Homozygous iPSCs differentiated to cortical excitatory neurons showed significant increases in voltage-dependent Na^+^ currents and intrinsic excitability that were both rescued by a reintroduction of the human wild-type PRRT2. The same phenotype was shared by primary cortical neurons from the PRRT2 KO mouse that also displayed increased Na^+^ currents and heightened spontaneous and evoked electrical activity at the single-cell and network levels.

By comparing the mouse model with the phenotype of iPS-derived neurons, the study demonstrates that PRRT2 is an important negative modulator of Na^+^ channels, and that the lack of PRRT2 leads to Na^+^ channel hyperactivity in human and mouse neurons.

The iPSC models of genetic epilepsies cited above are summarized in Table 1.

## 3. Recent Advances in iPSC Technology for Modeling Epilepsy

### 3.1. Improvements in 2D Culture: Single Cell Models to Study Synaptic Transmission

In the last years, several studies have been carried out to model epilepsy, and technological advances have been undertaken to improve the differentiation of human neurons from iPSCs in the context of a controlled genetic background [61,62,63]. Extensive analysis of morphology, polarity and the expression profile of iPSC-derived human neurons, as well as their firing properties and spontaneous synaptic activity, demonstrated the potential of this model in experimental epileptology [55,64,65,66]. On the other hand, iPSC-derived human neurons showed significant limits toward the dissection of relevant neuronal phenotypes, such as evoked synaptic transmission and short-term plasticity. Indeed, mass culturing protocols display major shortcomings: the heterogeneous maturation degree of iPSC-derived neuronal cultures resulted in an incomplete development of synaptic transmission and the high and uncontrolled complexity of the network.

Investigating epileptogenic mechanisms in cells requires quantitative measures of the synaptic input and output of individual human neurons. Autaptic cultures, in which single neurons grow in isolation and form synapses exclusively with themselves (autapses), represent valuable tools to study synaptic transmission compared to complex neuronal networks. Under this condition, a single patch-clamp electrode can stimulate the neuronal cell body and record evoked post-synaptic responses; moreover, the application of different stimulation protocols allows the analysis of different types of synaptic plasticity.

**Figure 1:**
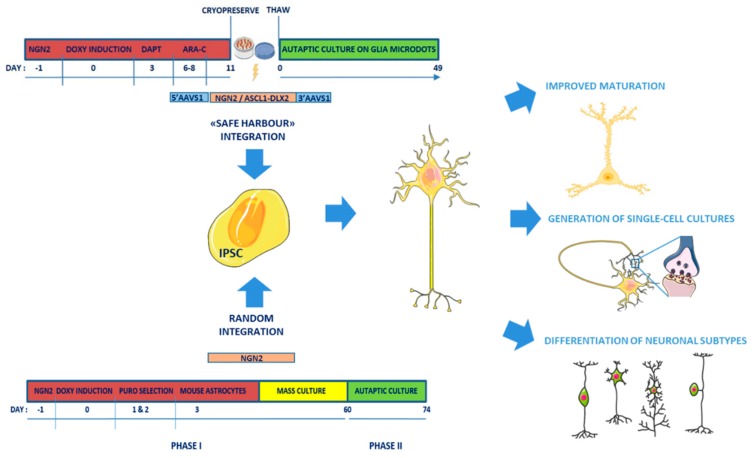
Single-cell approaches to generate mature human neurons from induced Pluripotent Stem Cells (iPSCs) to study synapse function. Overview of the new culture protocols (safe harbor integration [68,69] or random integration/two phase protocol [67]) for generating mature human neurons from iPSCs to study the morphological and functional parameters underlying human synaptic transmission in specific neuronal subtypes.

Recently, three independent research groups developed efficient protocols to improve the survival rate of human single-cell cultures and obtain human autaptic neurons (Figure 1) [67,68,69].

Fenske et al. [67] developed a protocol based on two-phases: in the first phase, human iPSCs are differentiated into mature neurons by exogenously expressing Neurogenin 2 (NGN2) [70], and maintained for 60 days in a mass culture setting; then mature neurons are re-plated as single cells onto astrocytic micro-islands for a second autaptic culturing phase lasting a further 2 weeks. The authors demonstrated that the mass phase promoting cell-to-cell contact is essential to reach the full maturation and high survival rate of autaptically-induced neurons. These autaptically-induced neurons showed passive membrane properties comparable to primary murine autapticneurons, and actioned the potential firing and expression of AMPA and GABA neurotransmitter receptors, whereas NMDA were poorly expressed. Spontaneous and evoked synaptic transmission was comparable to that of autaptic rodent neurons, and synaptic responses were much larger than those measured in mass culture human neurons. Synaptic properties, such as the count of the number of readily-releasable synaptic vesicles per synapse, rates of release probability and spontaneous release, could be now assessed in vitro in patient-derived neurons to dissect epileptogenic pathways resulting from specific gene mutations.

To improve single neuron cultures, Meijer et al. developed a forward programming protocol in which the inducible NGN2 cassette is targeted to the AAVS1 “genomic safe harbor” locus in the iPS cell line by Talen gene editing technologies [68]. This strategy is crucial to decrease any variability due to different expression levels and insertional mutagenesis, and allows rapid conversion of iPSCs into neurons [71,72]. Moreover, transient addition of DAPT, an inhibitor of Notch signaling, and Ara-C to synchronize cells to entry in a post-mitotic, neuronal state, allowed the generation of a pure, highly standardized neuronal culture within 11 days from NGN2 induction. Hence, induced neurons are frozen and then plated onto a rat astrocytic microdot array to generate single-cell neuronal cultures. After 3 weeks, most induced neurons showed spontaneous synaptic responses, and the frequency of mEPSCs increased during maturation (from 2–6 weeks). However, only after 5–6 weeks of differentiation, autaptic neurons showed mature synaptic transmission in terms of robust evoked AMPA- and NMDA-mediated responses and morphologically mature synapses, as stated by the colocalization of postsynaptic Homer1 with the presynaptic synapsin puncta.

These data demonstrate that the spontaneous event frequency, observed at 3 weeks of differentiation, is a poor predictor of synaptic maturity, because the ability of synapses to synchronize their evoked responses appears only at 6 weeks of differentiation, thus disproving a large fraction of published papers based only on the studies of spontaneous synaptic responses.

This work provides a robust and efficient protocol to analyze evoked responses, which are crucial for investigating synaptic development. In addition, these cultures allow the assessment of fundamental synaptic parameters for structural and functional plasticity, such as paired pulse-plasticity, synaptic depression and synaptic recovery, not only in glutamatergic-induced NGN2 neurons, but also in other neuronal subtypes, such as GABAergic neurons obtained with ad hoc differentiation protocols [73]. These autaptic human GABAergic neurons develop synapses between 6 and 8 weeks in culture, show evoked synaptic transmission and short-term plasticity, and can be used as a disease model for GABAergic synaptic dysfunctions.

The accompanying paper from Rhee et al. [69] focuses on a thorough analysis of the active and passive membrane properties of autaptic neurons, obtained by either classical differentiation or transcription factor-based forward programming, forcing the expression of NGN2 [68] or ASCL-1 and DLX2 into safe-harbor AAVS1 alleles [74,75] to generate glutamatergic or GABAergic forebrain neurons, respectively. Interestingly, the GABAergic neuronal population differentiated with this protocol includes different subtypes of GABAergic neurons, such as a high percentage of parvalbumin neurons and also calbindin and somatostatin subtypes, suggesting a higher level of the culture maturation compared to previous protocols. Mouse astrocytes in micro-island cultures and 0.5% fetal bovine serum (FBS) medium resulted the optimal combination to improve the cell survival and maturation of synaptic functionality. Both glutamatergic and GABAergic neurons, induced by forced expression of transcription factors, showed evoked postsynaptic currents and short-term plasticity similar to mouse cortical neurons and earlier than human neurons differentiated with classical protocols. This study demonstrates that the autaptic system circumvents heterogeneity among iPSC-derived neurons, and enables researchers to investigate impairments in synapse formation, transmission and plasticity.

The autaptic cell culture technique can also be combined with traditional methods to investigate calcium signaling and the synaptic vesicle cycle [76,77,78,79]. Additionally, the combination of electrophysiological analysis with single-cell RNA sequencing (scRNA-seq) in a Patch-Seq approach allows the identification of the molecular signaling pathways underlying a particular cellular phenotype associated with specific disorders [65,80,81,82]. A recent work by van den Hurk et al. described an innovative Patch-Seq protocol that allows the analysis of the whole neuron, including cytosolic RNA in the soma, as well as distant RNA in the axon and dendrites, providing an accurate representation of the whole transcriptomic profile and a picture of distal synaptic/dendritic mRNA trafficking [83]. Patch-Seq analysis enables multimodal correlation between gene expression profiles, physiological function and the morphology of single cells, and could be applied to profile the transcriptome of neurons that have been pre-characterized with patch-clamping to reduce the phenotypic variability and heterogeneity.

### 3.2. Improvement in 3D Culture: Organoids

Among the new technologies, the most promising way to model genetic epilepsy are the cerebral organoids. In the last three years, publications reporting the use of cerebral organoid raised exponentially. The cerebral organoids are 3D self-assembled structures obtained from human PSCs able to generate an organized architecture made of different kinds of cells and capable of functional behavior, recapitulating the human fetal brain [84,85,86].

At the state-of-the-art, the main works on syndromes involving epilepsy are strictly related to the neurodevelopment. They are focused on defective migration and cerebral malformations, that are features that cannot be addressed in a 2D-model of human neurons.

Blair et al. modeled *Tuberous sclerosis complex* (TSC) disorder [56]. They generated TSC2-/- cortical spheroids and found the alteration of cell number and morphology compared to controls. Furthermore, they tested the hypothesis of “second-hit”, by creating a Cre-inducible KO organoid model. This approach enables us to evaluate the role of timing and the size effect of the second hit. They found that biallelic inactivation alters the morphology, and that interestingly, the alterations are reversible by early treatment with rapamycin.

*Lissencephaly* is a brain malformation of heterogeneous genetic etiology associated with mental retardation and intractable epilepsy and characterized by nearly absent cortical folding. The use of cerebral organoids in this condition is particularly attractive, as the mouse model is physiologically lissencephalic. The Miller-Dieker syndrome is the most severe form, and is caused by the deletion of the 17p13.3 locus, that includes LIS1 and YWHAE and several additional genes. By live imaging and scRNA-seq, Bershteyn et al. identified altered neuronal migration and mitotic defects in the organoid’s outer radial glia, a population of cells in the outer subventricular zone responsible for the cortical upper-layer neurogenesis [57]. Similarly, Iefremova et al. identified a disruption of the cortical niche and altered the division of ventral zone radial glial cell, probably responsible for a decrease of organoid size (notably brain size reduction is a main feature of disease) [58]. In addition, they rescued the organoid phenotype by modulating the WNT pathway by the pharmacological activation of B-catenin. Lissencephaly is also caused by the mutation of LIS1. Reiner’s lab developed an “organoid on-chip” system able to provide nutrient exchange diffusion and in situ whole organ fluorescent live imaging over several weeks. Using this approach, they found in LIS1+/- organoids reduced convolution, altered elastic properties and cytoskeleton related genes [59].

*Timothy syndrome* is another neurodevelopmental disorder caused by point mutations in the CACNA1C gene. Birey et al. developed an assembloid, i.e., an organoid composed of two different spheroids resembling the dorsal and ventral forebrain, and generated the first human model of saltatory migration of interneurons to the cerebral cortex characterized by intermittent movement of soma [60]. Combining live cell imaging and scRNA-seq, they identified an abnormal saltatory behavior in the Cav 1.2 mutated organoid. In addition, they rescued the saltatory defects treating the assembloids with L-type calcium channels inhibitors (nimodipine).

### 3.3. New Advancement in Organoid Technology

#### 3.3.1. Electrophysiological Analysis

Trujillo et al. evaluated cerebral organoid extracellular spontaneous electrical activity using MEA and scRNA-seq to investigate both cellular identity and differentiation [87]. They demonstrated that cortical organoids show an enhanced and constant increase of electrical activity (mean firing rate, burst frequency and synchrony) over time (10 months) compared to iPSC 2D culture. More importantly, they discover for the first time that organoid models generate nested oscillatory network dynamics, where the phase of low frequency oscillation modulates the higher-frequency oscillatory pattern. In addition, by pharmacological inhibition, they showed the involvement of glutamatergic and GABAergic neurons in producing and maintaining oscillations. Collectively, these data suggest that this model is a valuable tool for studying the physiology of brain network formation during human development. Despite the recent advances in the understanding of the functional activity of organoids, this is the first evidence that cerebral organoids develop complex and functional neural network activity. In addition, electrophysiological recordings could be combined with optogenetic stimulation to better investigate networks dynamics and neural oscillations. For these reasons, this approach will be very useful to evaluate the spontaneous neural activity in models of genetic epilepsy and to evaluate drug efficacy [88].

#### 3.3.2. Reproducibility and Size of Cerebral Organoids

From the seminal works, one of the challenges of applying brain organoids to disease modeling and, ultimately, to large-scale drug screening, is the low reproducibility. During the last few years, much has been done to cope with this issue; in particular, Yoon et al. reached a very high degree of the reproducibility of cortical organoids by standardizing a feeder- and xeno-free method. This approach makes their methods usable for large-scale differentiation experiments and disease modeling [89]. At the state of the art, cerebral organoids are able to model only the early to middle stages of embryonal brain development, and indeed the small size of current organoids remains the fundamental limiting factor that reduces the possibility to fully recapitulate late stages of human brain development. The absence of a vascular system is also a real challenge in cerebral organoids; in fact, long-term organoid cultures consistently exhibit apoptotic cell death at the inner regions [90]. Therefore, these are the main technical limitations to study the mechanisms of epileptogenesis at late stage of neurodevelopment. For these reasons, many efforts are directed to accelerate organoids’ development or to increase organoids’ size. For instance, Cakir et al. engineered hESC to express the transcription factor ETS2 which is able to induce endothelial differentiation from fibroblasts [91]. ETS2 expression generated the formation of a vascular structure able to support oxygen diffusion, avoiding apoptosis of the inner part. This new tool creates a complex vascular-like network in cerebral organoids, making it a robust model to study brain disease *in vitro* for a long time.

#### 3.3.3. Next-Generation Imaging Technology

Many groups are developing genetically-encoded voltage and calcium indicators for functional live-cell imaging studies [92]. The last advancements have provided genetically-encoded indicators with a higher spatial and temporal resolution, solving the problem of the photostability of fluorescent proteins (the last generation indicators are Voltron and XCamp, respectively, for voltage and calcium measurements) [93,94].

These last generation indicators enable a non-invasive, long term, repetitive and unbiased functional imaging. Furthermore, they will be handy to monitor neuronal activity in live imaging during the high throughput screening of antiepileptic drugs.

**Figure 2:**
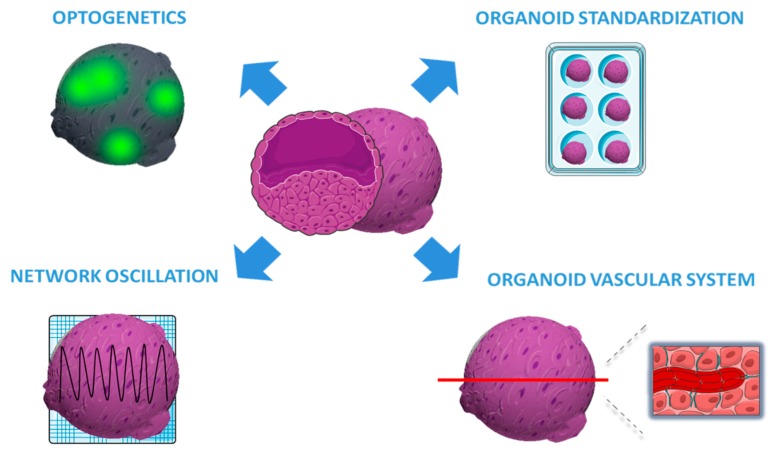
Recent advancements in organoid technologies. The figure illustrates the most recent advances in organoid technologies aimed at standardizing the organoid growth, enhancing vascularization, and improving the functional analysis by optogenetics and Multiple Electrode Array (MEA) electrophysiology.

The last advances in organoid technologies are schematized in Figure 2.

## 4. iPSC-Based Neural Transplantation for Genetic Disease Modeling

Transplantation of human iPSCs in rodent models emerged as an innovative methodology for disease modeling. The progress of xenotransplantation opened new perspectives for the generation of more complex iPSC-derived systems, called “chimeras” [95]. iPSC-derived cells and/or organoids could be transplanted into the CNS of rodent models. This will allow the differentiation and functioning of human cells into a physiological environment for the modeling of processes, such as defects in neuronal specification/migration and circuitry abnormalities, that may underlie different neurologic diseases [96,97]. A functional analysis of mutations affecting genes encoding for the Tuberous Sclerosis protein complex (TSC) provided significant insights into the potential of these approaches [47].

Nadadhur et al. tested the interaction of TSC neurons and TSC oligodendrocytes in an in vivo system, to evaluate functional effects under physiological conditions and for long periods [47]. To this aim, they transplanted control or TSC neuron-oligodendrocyte populations into the brains of immunocompromised mice. A massive incorporation of human cells types in the mouse brain at 2–3 months after transplantation was observed in many brain regions, such as the cerebral cortex, caudate putamen and the olfactory bulb. These in vivo experiments increase the complexity of the experimental system, thus allowing an extensive investigation of TSC phenotypes.

## 5. iPSC-Based Neural Transplantation for Non-Genetic Epilepsies: Temporal Lobe Epilepsies (TLE)

Temporal lobe epilepsy (TLE) is a common form of epilepsy characterized by recurrent focal, dyscognitive seizures originating from the temporal lobe [98]. The genetic etiology is recognized in rare familial forms and is unknown in most cases. TLE is often associated with mesial temporal sclerosis (MSTLE), characterized by the loss of GABAergic inhibitory interneurons and sprouting of granule cell axons (or mossy fibers) in the hippocampus [99,100,101].

Cortical interneurons represent a large component of local-circuit, GABA-releasing neurons, and constitute ~20% of the total neuron population in the human cerebral cortex [102,103]. In humans, cortical interneurons derive from two regions of the ventral lateral ventricle in the embryonic telencephalon, the medial and caudal ganglionic eminences (MGE and CGE), and from a defined region of the pre-optic area [104]. It has been shown that interneuron progenitor cells dissected from the embryonic MGE maintain their migratory properties after their graft into a rodent brain [105,106], providing the basis for the application of xenotransplantation approaches in the study of TLE.

Recapitulating TLE using classical 2D iPSC cultures is trivial due to the lack of information on the etiology and the intense circuitry re-organization processes underlying this common epilepsy. In this scenario, iPSC-derived grafts within in vivo models emerged as a promising approach to investigate the pathophysiology of TLE [107,108,109].

Toward the modeling of TLE, Avaliani et al. evaluated the effect of human iPSC-derived neural progenitor grafts in an in vivo rodent model, to clarify the synaptic communication between grafted iPSC-derived neurons and host neurons, which was never assessed in previous studies. They showed that the host-to-host graft synaptogenesis in transplanted iPSC-derived cells took place in about 6 months, together with the maturation of a functional neuronal phenotype with a prevalence of GABA and GAD positive neurons [110]. Here, the authors demonstrated that application of human iPSC-derived cells grafts represents a powerful approach to (i) drive through autologous MGE cell grafting; (ii) achieve seizure control; (iii) avoid any ethical limitation related to the use of animal models and iv) reduce the long-term immune suppression.

**Figure 3:**
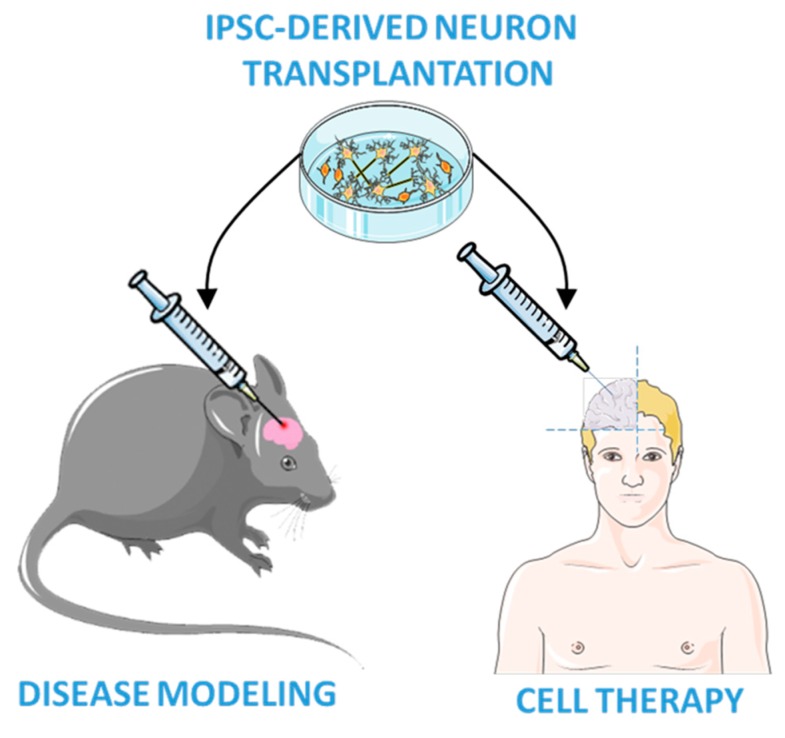
iPSC-based transplantation technologies. iPSC-derived neuroprecursors are transplanted into an adult mouse brain to study neuronal differentiation for epilepsy modeling and for future cell therapies in humans.

Following this preliminary work, Upadhya and collaborators performed iPSCs-derived neural cell grafts into the hippocampus of a standard rat model of chronic TLE [111], i.e., the intra-hippocampal injection of kainic acid to induce *status epilepticus* and trigger spontaneous recurrent seizures. They further develop methods to improve the in vivo differentiation and functional integration of iPSC neurons by using iPSC-derived MGE-like interneuron precursors [112]. The efficient differentiation of iPSC-engrafted cells into GABAergic neurons after *status epilepticus* preserved the local inhibitory circuitry of the hippocampus and mitigated the progression of *status epilepticus*-induced injury. These findings supported that transplanted stem cells are safe, and may efficiently reduce seizures in rodent models, providing evidence of the potential therapeutic efficacy of the iPSC-based therapy in epilepsy, as illustrated in Figure 3. However, iPSC graft techniques are still in their infancy, thus additional evidences are required to bring them into clinical practice.

## 6. Conclusions

IPSC-derived neurons, generated by patients affected by epilepsy, represent a powerful model to investigate the disease pathophysiology, and have contributed to the understanding of the molecular mechanisms of the epileptic phenotypes. The most recent advances in iPSC-based technologies are very promising for epilepsy research. The single autaptic neuron cultures allow now a reliable characterization of synaptic transmission and plasticity, providing the possibility of a deeper characterization of the epileptic mechanisms. The efforts in the standardization of cerebral organoids are opening the road to the 3D characterization of the neural circuits. Finally, the iPSC transplantation is more challenging, but represents an original and efficient approach to analyze genetic and non-genetic epilepsies, and may be attractive for future cell-therapies.

## Figures and Tables

**Table 1:** Summary of iPSC-based models of epilepsy.

Syndromes	Gene	Model	iPSCs and Mutations	Main Results	References
**Epileptic Encephalopathies**					
Dravet	SCN1A	2D	iPSCs from patients (IV14 + 3A > T splice site mutation, Y325X [20] p.F1414I [21])	Hyperexcitability of excitatory and inhibitory neurons	[20,21]
Dravet	SCN1A	2D	iPSCs from patient (p.R1645 Ter [22]; p.Q1923R [23]; p.G1410W [25])	Hypoexcitability of inhibitory neurons	[22,23,25]
Dravet	SCN1A	2D	iPSCs from patient: p.S1328P	Hypoexcitability of inhibitory neurons, no phenotype for excitatory	[26]
Dravet	SCN1A	2D	iPSCs from two patients (p.P1837Rfs24; p.A989P)	Transcriptome alterations in inhibitory neurons	[24]
MMPSI	KCNT1	2D	iPSCs from patients (heterozygous p.P924L)	Neuronal hyperexcitability in forebrain neurons	[32]
PCDH19-GCE	PCDH19	2D	iPSCs from a healthy control	PCDH19 role in apicobasal polarity of neuroprogenitor cells	[39]
PCDH19-GCE	PCDH19	2D	iPSCs from patients (p.N377H and p.I557N)	Loss of polarity and increased neurogenesis of neural stem (NSC) and progenitor cells	[38]
**Malformative epilepsy**					
TSC disorder	TSC	2D	ESC line SA001 (gene editing to obtain TSC-/-)	Altered synaptogenesis and synaptic transmission in NSCs and mature neurons	[46]
TSC disorder	TSC	2D	ESC line SA001 (gene editing to obtain TSC-/-)	Active inflammation response and metabolic activity in NSC of TSC-/-	[44]
TSC disorder	TSC	2D	iPSCs from TSC2+/- patient (c.4650_4653del)	Delayed neuronal differentiation	[45]
TSC disorder	TSC	2D	iPSCs from TSC2+/- patients (p.W750X and p.H522T)	Increased network activity; neuron–glia co-cultures increase neuronal defects	[47]
TSC disorder	TSC	2D	iPSCs from TSC2+/- patients (18bp del_exon 41); gene editing to obtain TSC-/-	TSC-/- and TSC+/- show neuronal morphological abnormalities; increased neuronal activity of TSC-/- neurons	[48]
TSC disorder	TSC	3D	iPSCs TSC2+/- patient (del ex1-14); gene editing to obtain conditional TSC-/-	Biallelic inactivation is responsible for the formation of dysplastic cells and gliosis in 3D cortical spheroids	[56]
Miller-Dieker syndrome (MDS)	17p13.3 deletion	3D	iPSCs from patients (deletion of 17p13.3)	Altered neuronal migration and mitotic defects in outer radial glial cells	[57]
Miller-Dieker syndrome (MDS)	17p13.3 deletion	3D	iPSCs from patients (deletion of 17p13.3)	Decreased cerebral organoid size and mitotic defect in ventral zone radial glial cell	[58]
Lissencephaly	LIS1	3D	NIHhESC-10-0079 (gene editing to obtain LIS1+/-)	Reduced convolution, altered elastic properties and cytoskeleton-related genes	[59]
Lissencephaly	TUBA1A	2D	iPSCs from patients (p.N329S and p.R264C)	Reduced neurite extension from the neurospheres of neural progenitor cells	[51]
Timothy syndrome	CACNA1C	3D	iPSCs from patients (p.G406R)	Abnormal saltatory migration of interneurons in cerebral assembloids	[60]
**Idiopathic Epilepsy**					
BFIE	PRRT2	2D	iPSCs from patients (p.R217Pfs8)	Hyperexcitability of cortical excitatory neurons	[55]

## References

[1] Takahashi K., Tanabe K., Ohnuki M., Narita M., Ichisaka T., Tomoda K., Yamanaka S. (2007). Induction of Pluripotent Stem Cells from Adult Human Fibroblasts by Defined Factors. Cell.

[2] Ghaffari L.T., Starr A., Nelson A.T., Sattler R. (2018). Representing Diversity in the Dish: Using Patient-Derived in Vitro Models to Recreate the Heterogeneity of Neurological Disease. Front. Neurosci..

[3] Mertens J., Reid D., Lau S., Kim Y., Gage F.H. (2018). Aging in a Dish: iPSC-Derived and Directly Induced Neurons for Studying Brain Aging and Age-Related Neurodegenerative Diseases. Annu. Rev. Genet..

[4] Soliman M.A., Aboharb F., Zeltner N., Studer L. (2017). Pluripotent stem cells in neuropsychiatric disorders. Mol. Psychiatry.

[5] Niu W., Parent J.M. (2019). Modeling genetic epilepsies in a dish. Dev. Dyn..

[6] Shi Y., Inoue H., Wu J.C., Yamanaka S. (2017). Induced pluripotent stem cell technology: A decade of progress. Nat. Rev. Drug Discov..

[7] Hockemeyer D., Jaenisch R. (2016). Induced Pluripotent Stem Cells Meet Genome Editing. Cell Stem Cell.

[8] Nolan D., Fink J. (2018). Genetics of epilepsy. Handbook of Clinical Neurology.

[9] Holmes G.L., Noebels J.L. (2016). The Epilepsy Spectrum: Targeting Future Research Challenges. Cold Spring Harb. Perspect. Med..

[10] Dravet C., Bureau M., Bernardina B.D., Guerrini R. (2011). Severe myoclonic epilepsy in infancy (Dravet syndrome) 30 years later. Epilepsia.

[11] Catterall W.A., Kalume F., Oakley J.C. (2010). Na _V_ 1.1 channels and epilepsy. J. Physiol..

[12] Escayg A., Goldin A.L. (2010). Sodium channel SCN1A and epilepsy: Mutations and mechanisms. Epilepsia.

[13] Yu F.H., Mantegazza M., Westenbroek R.E., Robbins C.A., Kalume F., Burton K.A., Spain W.J., McKnight G.S., Scheuer T., Catterall W.A. (2006). Reduced sodium current in GABAergic interneurons in a mouse model of severe myoclonic epilepsy in infancy. Nat. Neurosci..

[14] Cheah C.S., Yu F.H., Westenbroek R.E., Kalume F.K., Oakley J.C., Potter G.B., Rubenstein J.L., Catterall W.A. (2012). Specific deletion of NaV1.1 sodium channels in inhibitory interneurons causes seizures and premature death in a mouse model of Dravet syndrome. Proc. Natl. Acad. Sci. USA.

[15] Dutton S.B., Makinson C.D., Papale L.A., Shankar A., Balakrishnan B., Nakazawa K., Escayg A. (2013). Preferential inactivation of Scn1a in parvalbumin interneurons increases seizure susceptibility. Neurobiol. Dis..

[16] Ogiwara I., Miyamoto H., Morita N., Atapour N., Mazaki E., Inoue I., Takeuchi T., Itohara S., Yanagawa Y., Obata K. (2007). Nav1.1 Localizes to Axons of Parvalbumin-Positive Inhibitory Interneurons: A Circuit Basis for Epileptic Seizures in Mice Carrying an Scn1a Gene Mutation. J. Neurosci..

[17] Tai C., Abe Y., Westenbroek R.E., Scheuer T., Catterall W.A. (2014). Impaired excitability of somatostatin- and parvalbumin-expressing cortical interneurons in a mouse model of Dravet syndrome. Proc. Natl. Acad. Sci. USA.

[18] Favero M., Sotuyo N.P., Lopez E., Kearney J.A., Goldberg E.M. (2018). A Transient Developmental Window of Fast-Spiking Interneuron Dysfunction in a Mouse Model of Dravet Syndrome. J. Neurosci..

[19] Mistry A.M., Thompson C.H., Miller A.R., Vanoye C.G., George A.L., Kearney J.A. (2014). Strain- and age-dependent hippocampal neuron sodium currents correlate with epilepsy severity in Dravet syndrome mice. Neurobiol. Dis..

[20] Liu Y., Lopez-Santiago L.F., Yuan Y., Jones J.M., Zhang H., O’Malley H.A., Patino G.A., O’Brien J.E., Rusconi R., Gupta A. (2013). Dravet syndrome patient-derived neurons suggest a novel epilepsy mechanism. Ann. Neurol..

[21] Jiao J., Yang Y., Shi Y., Chen J., Gao R., Fan Y., Yao H., Liao W., Sun X.-F., Gao S. (2013). Modeling Dravet syndrome using induced pluripotent stem cells (iPSCs) and directly converted neurons. Hum. Mol. Genet..

[22] Higurashi N., Uchida T., Lossin C., Misumi Y., Okada Y., Akamatsu W., Imaizumi Y., Zhang B., Nabeshima K., Mori M.X. (2013). A human Dravet syndrome model from patient induced pluripotent stem cells. Mol. Brain.

[23] Liu J., Gao C., Chen W., Ma W., Li X., Shi Y., Zhang H., Zhang L., Long Y., Xu H. (2016). CRISPR/Cas9 facilitates investigation of neural circuit disease using human iPSCs: Mechanism of epilepsy caused by an SCN1A loss-of-function mutation. Transl. Psychiatry.

[24] Schuster J., Laan L., Klar J., Jin Z., Huss M., Korol S., Noraddin F.H., Sobol M., Birnir B., Dahl N. (2019). Transcriptomes of Dravet syndrome iPSC derived GABAergic cells reveal dysregulated pathways for chromatin remodeling and neurodevelopment. Neurobiol. Dis..

[25] Kim H.W., Quan Z., Kim Y.B., Cheong E., Kim H.D., Cho M., Jang J., Yoo Y.R., Lee J.S., Kim J.H. (2018). Differential effects on sodium current impairments by distinct SCN1A mutations in GABAergic neurons derived from Dravet syndrome patients. Brain Dev..

[26] Sun Y., Paşca S.P., Portmann T., Goold C., Worringer K.A., Guan W., Chan K.C., Gai H., Vogt D., Chen Y.J.J. (2016). A deleterious Nav1.1 mutation selectively impairs telencephalic inhibitory neurons derived from Dravet Syndrome patients. Elife.

[27] Sun Y., Dolmetsch R.E. (2018). Investigating the Therapeutic Mechanism of Cannabidiol in a Human Induced Pluripotent Stem Cell (iPSC)-Based Model of Dravet Syndrome. Cold Spring Harb. Symp. Quant. Biol..

[28] Barcia G., Fleming M.R., Deligniere A., Gazula V.-R., Brown M.R., Langouet M., Chen H., Kronengold J., Abhyankar A., Cilio R. (2012). De novo gain-of-function KCNT1 channel mutations cause malignant migrating partial seizures of infancy. Nat. Genet..

[29] Kim G.E., Kaczmarek L.K. (2014). Emerging role of the KCNT1 Slack channel in intellectual disability. Front. Cell. Neurosci..

[30] Dilena R., DiFrancesco J.C., Soldovieri M.V., Giacobbe A., Ambrosino P., Mosca I., Galli M.A., Guez S., Fumagalli M., Miceli F. (2018). Early Treatment with Quinidine in 2 Patients with Epilepsy of Infancy with Migrating Focal Seizures (EIMFS) Due to Gain-of-Function KCNT1 Mutations: Functional Studies, Clinical Responses, and Critical Issues for Personalized Therapy. Neurotherapeutics.

[31] Bausch A.E., Dieter R., Nann Y., Hausmann M., Meyerdierks N., Kaczmarek L.K., Ruth P., Lukowski R. (2015). The sodium-activated potassium channel Slack is required for optimal cognitive flexibility in mice. Learn. Mem..

[32] Quraishi I.H., Stern S., Mangan K.P., Zhang Y., Ali S.R., Mercier M.R., Marchetto M.C., McLachlan M.J., Jones E.M., Gage F.H. (2019). An Epilepsy-Associated KCNT1 Mutation Enhances Excitability of Human iPSC-Derived Neurons by Increasing Slack K _Na_ Currents. J. Neurosci..

[33] Kolc K.L., Sadleir L.G., Scheffer I.E., Ivancevic A., Roberts R., Pham D.H., Gecz J. (2019). A systematic review and meta-analysis of 271 PCDH19-variant individuals identifies psychiatric comorbidities, and association of seizure onset and disease severity. Mol. Psychiatry.

[34] Trivisano M., Specchio N. (2019). The role of PCDH19 in refractory status epilepticus. Epilepsy Behav..

[35] Pederick D.T., Homan C.C., Jaehne E.J., Piltz S.G., Haines B.P., Baune B.T., Jolly L.A., Hughes J.N., Gecz J., Thomas P.Q. (2016). Pcdh19 Loss-of-Function Increases Neuronal Migration In Vitro but is Dispensable for Brain Development in Mice. Sci. Rep..

[36] Pederick D.T., Richards K.L., Piltz S.G., Kumar R., Mincheva-Tasheva S., Mandelstam S.A., Dale R.C., Scheffer I.E., Gecz J., Petrou S. (2018). Abnormal Cell Sorting Underlies the Unique X-Linked Inheritance of PCDH19 Epilepsy. Neuron.

[37] Hayashi S., Inoue Y., Hattori S., Kaneko M., Shioi G., Miyakawa T., Takeichi M. (2017). Loss of X-linked Protocadherin-19 differentially affects the behavior of heterozygous female and hemizygous male mice. Sci. Rep..

[38] Homan C.C., Pederson S., To T.-H., Tan C., Piltz S., Corbett M.A., Wolvetang E., Thomas P.Q., Jolly L.A., Gecz J. (2018). PCDH19 regulation of neural progenitor cell differentiation suggests asynchrony of neurogenesis as a mechanism contributing to PCDH19 Girls Clustering Epilepsy. Neurobiol. Dis..

[39] Compagnucci C., Petrini S., Higuraschi N., Trivisano M., Specchio N., Hirose S., Bertini E., Terracciano A. (2015). Characterizing PCDH19 in human induced pluripotent stem cells (iPSCs) and iPSC-derived developing neurons: Emerging role of a protein involved in controlling polarity during neurogenesis. Oncotarget.

[40] Curatolo P., Cusmai R., Cortesi F., Chiron C., Jambaque I., Dulac O. (1991). Neuropsychiatric Aspects of Tuberous Sclerosis. Ann. N.Y. Acad. Sci..

[41] Crino P.B., Aronica E., Baltuch G., Nathanson K.L. (2010). Biallelic TSC gene inactivation in tuberous sclerosis complex. Neurology.

[42] Lim J.S., Gopalappa R., Kim S.H., Ramakrishna S., Lee M., Kim W., Kim J., Park S.M., Lee J., Oh J.H. (2017). Somatic Mutations in TSC1 and TSC2 Cause Focal Cortical Dysplasia. Am. J. Hum. Genet..

[43] Crino P.B. (2013). Evolving neurobiology of tuberous sclerosis complex. Acta Neuropathol..

[44] Grabole N., Zhang J.D., Aigner S., Ruderisch N., Costa V., Weber F.C., Theron M., Berntenis N., Spleiss O., Ebeling M. (2016). Genomic analysis of the molecular neuropathology of tuberous sclerosis using a human stem cell model. Genome Med..

[45] Zucco A.J., Pozzo V.D., Afinogenova A., Hart R.P., Devinsky O., D’Arcangelo G. (2018). Neural progenitors derived from Tuberous Sclerosis Complex patients exhibit attenuated PI3K/AKT signaling and delayed neuronal differentiation. Mol. Cell. Neurosci..

[46] Costa V., Aigner S., Vukcevic M., Sauter E., Behr K., Ebeling M., Dunkley T., Friedlein A., Zoffmann S., Meyer C.A. (2016). mTORC1 Inhibition Corrects Neurodevelopmental and Synaptic Alterations in a Human Stem Cell Model of Tuberous Sclerosis. Cell Rep..

[47] Nadadhur A.G., Alsaqati M., Gasparotto L., Cornelissen-Steijger P., van Hugte E., Dooves S., Harwood A.J., Heine V.M. (2019). Neuron-Glia Interactions Increase Neuronal Phenotypes in Tuberous Sclerosis Complex Patient iPSC-Derived Models. Stem Cell Rep..

[48] Winden K.D., Sundberg M., Yang C., Wafa S.M.A., Dwyer S., Chen P.F., Buttermore E.D., Sahin M. (2019). Biallelic Mutations in *TSC2* Lead to Abnormalities Associated with Cortical Tubers in Human iPSC-Derived Neurons. J. Neurosci..

[49] Keays D.A., Tian G., Poirier K., Huang G.-J., Siebold C., Cleak J., Oliver P.L., Fray M., Harvey R.J., Molnár Z. (2007). Mutations in α-Tubulin Cause Abnormal Neuronal Migration in Mice and Lissencephaly in Humans. Cell.

[50] Belvindrah R., Natarajan K., Shabajee P., Bruel-Jungerman E., Bernard J., Goutierre M., Moutkine I., Jaglin X.H., Savariradjane M., Irinopoulou T. (2017). Mutation of the α-tubulin Tuba1a leads to straighter microtubules and perturbs neuronal migration. J. Cell Biol..

[51] Bamba Y., Shofuda T., Kato M., Pooh R.K., Tateishi Y., Takanashi J., Utsunomiya H., Sumida M., Kanematsu D., Suemizu H. (2016). In vitro characterization of neurite extension using induced pluripotent stem cells derived from lissencephaly patients with TUBA1A missense mutations. Mol. Brain.

[52] Rossi P., Sterlini B., Castroflorio E., Marte A., Onofri F., Valtorta F., Maragliano L., Corradi A., Benfenati F. (2018). A novel topology of proline-rich transmembrane protein 2 (PRRT2): Hints for an intracellular function at the synapse. J. Biol. Chem..

[53] Valente P., Castroflorio E., Rossi P., Fadda M., Sterlini B., Cervigni R.I., Prestigio C., Giovedì S., Onofri F., Mura E. (2016). PRRT2 Is a Key Component of the Ca 2+ -Dependent Neurotransmitter Release Machinery. Cell Rep..

[54] Michetti C., Castroflorio E., Marchionni I., Forte N., Sterlini B., Binda F., Fruscione F., Baldelli P., Valtorta F., Zara F. (2017). The PRRT2 knockout mouse recapitulates the neurological diseases associated with PRRT2 mutations. Neurobiol. Dis..

[55] Fruscione F., Valente P., Sterlini B., Romei A., Baldassari S., Fadda M., Prestigio C., Giansante G., Sartorelli J., Rossi P. (2018). PRRT2 controls neuronal excitability by negatively modulating Na+ channel 1.2/1.6 activity. Brain.

[56] Blair J.D., Hockemeyer D., Bateup H.S. (2018). Genetically engineered human cortical spheroid models of tuberous sclerosis. Nat. Med..

[57] Bershteyn M., Nowakowski T.J., Pollen A.A., Di Lullo E., Nene A., Wynshaw-Boris A., Kriegstein A.R. (2017). Human iPSC-Derived Cerebral Organoids Model Cellular Features of Lissencephaly and Reveal Prolonged Mitosis of Outer Radial Glia. Cell Stem Cell.

[58] Iefremova V., Manikakis G., Krefft O., Jabali A., Weynans K., Wilkens R., Marsoner F., Brändl B., Müller F.J., Koch P. (2017). An Organoid-Based Model of Cortical Development Identifies Non-Cell-Autonomous Defects in Wnt Signaling Contributing to Miller-Dieker Syndrome. Cell Rep..

[59] Karzbrun E., Kshirsagar A., Cohen S.R., Hanna J.H., Reiner O. (2018). Human brain organoids on a chip reveal the physics of folding. Nat. Phys..

[60] Birey F., Andersen J., Makinson C.D., Islam S., Wei W., Huber N., Fan H.C., Metzler K.R.C., Panagiotakos G., Thom N. (2017). Assembly of functionally integrated human forebrain spheroids. Nature.

[61] Chambers S.M., Fasano C.A., Papapetrou E.P., Tomishima M., Sadelain M., Studer L. (2009). Highly efficient neural conversion of human ES and iPS cells by dual inhibition of SMAD signaling. Nat. Biotechnol..

[62] Gunhanlar N., Shpak G., van der Kroeg M., Gouty-Colomer L.A., Munshi S.T., Lendemeijer B., Ghazvini M., Dupont C., Hoogendijk W.J.G., Gribnau J. (2018). A simplified protocol for differentiation of electrophysiologically mature neuronal networks from human induced pluripotent stem cells. Mol. Psychiatry.

[63] Shi Y., Kirwan P., Livesey F.J. (2012). Directed differentiation of human pluripotent stem cells to cerebral cortex neurons and neural networks. Nat. Protoc..

[64] Nadadhur A.G., Emperador Melero J., Meijer M., Schut D., Jacobs G., Li K.W., Hjorth J.J.J., Meredith R.M., Toonen R.F., Van Kesteren R.E. (2017). Multi-level characterization of balanced inhibitory-excitatory cortical neuron network derived from human pluripotent stem cells. PLoS ONE.

[65] Bardy C., van den Hurk M., Kakaradov B., Erwin J.A., Jaeger B.N., Hernandez R.V., Eames T., Paucar A.A., Gorris M., Marchand C. (2016). Predicting the functional states of human iPSC-derived neurons with single-cell RNA-seq and electrophysiology. Mol. Psychiatry.

[66] Tang H., Sha H., Sun H., Wu X., Xie L., Wang P., Xu C., Larsen C., Zhang H.L., Gong Y. (2013). Tracking induced pluripotent stem cells-derived neural stem cells in the central nervous system of rats and monkeys. Cell. Reprogram..

[67] Fenske P., Grauel M.K., Brockmann M.M., Dorrn A.L., Trimbuch T., Rosenmund C. (2019). Autaptic cultures of human induced neurons as a versatile platform for studying synaptic function and neuronal morphology. Sci. Rep..

[68] Meijer M., Rehbach K., Brunner J.W., Classen J.A., Lammertse H.C.A., van Linge L.A., Schut D., Krutenko T., Hebisch M., Cornelisse L.N. (2019). A Single-Cell Model for Synaptic Transmission and Plasticity in Human iPSC-Derived Neurons. Cell Rep..

[69] Rhee H.J., Shaib A.H., Rehbach K., Lee C., Seif P., Thomas C., Gideons E., Guenther A., Krutenko T., Hebisch M. (2019). An Autaptic Culture System for Standardized Analyses of iPSC-Derived Human Neurons. Cell Rep..

[70] Zhang Y., Pak C., Han Y., Ahlenius H., Zhang Z., Chanda S., Marro S., Patzke C., Acuna C., Covy J. (2013). Rapid single-step induction of functional neurons from human pluripotent stem cells. Neuron.

[71] Pawlowski M., Ortmann D., Bertero A., Tavares J.M., Pedersen R.A., Vallier L., Kotter M.R.N. (2017). Inducible and Deterministic Forward Programming of Human Pluripotent Stem Cells into Neurons, Skeletal Myocytes, and Oligodendrocytes. Stem Cell Rep..

[72] Wang C., Ward M.E., Chen R., Liu K., Tracy T.E., Chen X., Xie M., Sohn P.D., Ludwig C., Meyer-Franke A. (2017). Scalable Production of iPSC-Derived Human Neurons to Identify Tau-Lowering Compounds by High-Content Screening. Stem Cell Rep..

[73] Liu Y., Liu H., Sauvey C., Yao L., Zarnowska E.D., Zhang S.C. (2013). Directed differentiation of forebrain GABA interneurons from human pluripotent stem cells. Nat. Protoc..

[74] Yang N., Chanda S., Marro S., Ng Y.H., Janas J.A., Haag D., Ang C.E., Tang Y., Flores Q., Mall M. (2017). Generation of pure GABAergic neurons by transcription factor programming. Nat. Methods.

[75] Qian K., Huang C.T., Chen H., Blackbourn L.W., Chen Y., Cao J., Yao L., Sauvey C., Du Z., Zhang S.C. (2014). A simple and efficient system for regulating gene expression in human pluripotent stem cells and derivatives. Stem Cells.

[76] Mizuno G.O., Wang Y., Shi G., Wang Y., Sun J., Papadopoulos S., Broussard G.J., Unger E.K., Deng W., Weick J. (2018). Aberrant Calcium Signaling in Astrocytes Inhibits Neuronal Excitability in a Human Down Syndrome Stem Cell Model. Cell Rep..

[77] Rost B.R., Schneider F., Grauel M.K., Wozny C., Bentz C., Blessing A., Rosenmund T., Jentsch T.J., Schmitz D., Hegemann P. (2015). Optogenetic acidification of synaptic vesicles and lysosomes. Nat. Neurosci..

[78] Egashira Y., Takase M., Watanabe S., Ishida J., Fukamizu A., Kaneko R., Yanagawa Y., Takamori S. (2016). Unique pH dynamics in GABAergic synaptic vesicles illuminates the mechanism and kinetics of GABA loading. Proc. Natl. Acad. Sci. USA.

[79] Herman M.A., Trimbuch T., Rosenmund C. (2018). Differential pH Dynamics in Synaptic Vesicles from Intact Glutamatergic and GABAergic Synapses. Front. Synaptic. Neurosci..

[80] Cadwell C.R., Palasantza A., Jiang X., Berens P., Deng Q., Yilmaz M., Reimer J., Shen S., Bethge M., Tolias K.F. (2016). Electrophysiological, transcriptomic and morphologic profiling of single neurons using Patch-seq. Nat. Biotechnol..

[81] Chen X., Zhang K., Zhou L., Gao X., Wang J., Yao Y., He F., Luo Y., Yu Y., Li S. (2016). Coupled electrophysiological recording and single cell transcriptome analyses revealed molecular mechanisms underlying neuronal maturation. Protein Cell.

[82] Cadwell C.R., Scala F., Li S., Livrizzi G., Shen S., Sandberg R., Jiang X., Tolias A.S. (2017). Multimodal profiling of single-cell morphology, electrophysiology, and gene expression using Patch-seq. Nat. Protoc..

[83] van den Hurk M., Erwin J.A., Yeo G.W., Gage F.H., Bardy C. (2018). Patch-Seq Protocol to analyze the electrophysiology, morphology and transcriptome of whole single neurons derived from Human pluripotent Stem Cells. Front. Mol. Neurosci..

[84] Benito-Kwiecinski S., Lancaster M.A. (2019). Brain Organoids: Human Neurodevelopment in a Dish. Cold Spring Harb. Perspect. Biol..

[85] Trujillo C.A., Muotri A.R. (2018). Brain Organoids and the Study of Neurodevelopment. Trends Mol. Med..

[86] Qian X., Song H., Ming G. (2019). Brain organoids: Advances, applications and challenges. Development.

[87] Trujillo C.A., Gao R., Negraes P.D., Gu J., Buchanan J., Preissl S., Wang A., Wu W., Haddad G.G., Chaim I.A. (2019). Complex Oscillatory Waves Emerging from Cortical Organoids Model Early Human Brain Network Development. Cell Stem Cell.

[88] Shiri Z., Lévesque M., Etter G., Manseau F., Williams S., Avoli M. (2017). Optogenetic Low-Frequency Stimulation of Specific Neuronal Populations Abates Ictogenesis. J. Neurosci..

[89] Yoon S.J., Elahi L.S., Pașca A.M., Marton R.M., Gordon A., Revah O., Miura Y., Walczak E.M., Holdgate G.M., Fan H.C. (2019). Reliability of human cortical organoid generation. Nat. Methods.

[90] Lancaster M.A., Knoblich J.A. (2014). Generation of cerebral organoids from human pluripotent stem cells. Nat. Protoc..

[91] Cakir B., Xiang Y., Tanaka Y., Kural M.H., Parent M., Kang Y.J., Chapeton K., Patterson B., Yuan Y., He C.S. (2019). Engineering of human brain organoids with a functional vascular-like system. Nat. Methods.

[92] Bando Y., Grimm C., Cornejo V.H., Yuste R. (2019). Genetic voltage indicators. BMC Biol..

[93] Abdelfattah A.S., Kawashima T., Singh A., Novak O., Liu H., Shuai Y., Huang Y.C., Campagnola L., Seeman S.C., Yu J. (2019). Bright and photostable chemigenetic indicators for extended in vivo voltage imaging. Science.

[94] Inoue M., Takeuchi A., Manita S., Horigane S., Sakamoto M., Kawakami R., Yamaguchi K., Otomo K., Yokoyama H., Kim R. (2019). Rational Engineering of XCaMPs, a Multicolor GECI Suite for In Vivo Imaging of Complex Brain Circuit Dynamics. Cell.

[95] Costamagna G., Andreoli L., Corti S., Faravelli I. (2019). iPSCs-Based Neural 3D Systems: A Multidimensional Approach for Disease Modeling and Drug Discovery. Cells.

[96] Barker R.A., Parmar M., Studer L., Takahashi J. (2017). Human Trials of Stem Cell-Derived Dopamine Neurons for Parkinson’s Disease: Dawn of a New Era. Cell Stem Cell.

[97] Espuny-Camacho I., Michelsen K.A., Gall D., Linaro D., Hasche A., Bonnefont J., Bali C., Orduz D., Bilheu A., Herpoel A. (2013). Pyramidal neurons derived from human pluripotent stem cells integrate efficiently into mouse brain circuits in vivo. Neuron.

[98] Scharfman H.E. (2007). The neurobiology of epilepsy. Curr. Neurol. Neurosci. Rep..

[99] de Lanerolle N.C., Kim J.H., Robbins R.J., Spencer D.D. (1989). Hippocampal interneuron loss and plasticity in human temporal lobe epilepsy. Brain Res..

[100] Malmgren K., Thom M. (2012). Hippocampal sclerosis--origins and imaging. Epilepsia.

[101] Zhang W., Buckmaster P.S. (2009). Dysfunction of the dentate basket cell circuit in a rat model of temporal lobe epilepsy. J. Neurosci..

[102] Freund T.F., Buzsáki G. (1996). Interneurons of the hippocampus. Hippocampus.

[103] Ascoli G.A., Alonso-Nanclares L., Anderson S.A., Barrionuevo G., Benavides-Piccione R., Burkhalter A., Buzsáki G., Cauli B., Defelipe J., Petilla Interneuron Nomenclature Group (2008). Petilla terminology: Nomenclature of features of GABAergic interneurons of the cerebral cortex. Nat. Rev. Neurosci..

[104] Gelman D., Griveau A., Dehorter N., Teissier A., Varela C., Pla R., Pierani A., Marín O. (2011). A wide diversity of cortical GABAergic interneurons derives from the embryonic preoptic area. J. Neurosci..

[105] Wichterle H., Turnbull D.H., Nery S., Fishell G., Alvarez-Buylla A. (2001). In utero fate mapping reveals distinct migratory pathways and fates of neurons born in the mammalian basal forebrain. Development.

[106] Alvarez-Dolado M., Calcagnotto M.E., Karkar K.M., Southwell D.G., Jones-Davis D.M., Estrada R.C., Rubenstein J.L., Alvarez-Buylla A., Baraban S.C. (2006). Cortical inhibition modified by embryonic neural precursors grafted into the postnatal brain. J. Neurosci..

[107] Oki K., Tatarishvili J., Wood J., Koch P., Wattananit S., Mine Y., Monni E., Tornero D., Ahlenius H., Ladewig J. (2012). Human-induced pluripotent stem cells form functional neurons and improve recovery after grafting in stroke-damaged brain. Stem Cells.

[108] Tornero D., Wattananit S., Grønning Madsen M., Koch P., Wood J., Tatarishvili J., Mine Y., Ge R., Monni E., Devaraju K. (2013). Human induced pluripotent stem cell-derived cortical neurons integrate in stroke-injured cortex and improve functional recovery. Brain.

[109] Nicholas C.R., Chen J., Tang Y., Southwell D.G., Chalmers N., Vogt D., Arnold C.M., Chen Y.J., Stanley E.G., Elefanty A.G. (2013). Functional maturation of hPSC-derived forebrain interneurons requires an extended timeline and mimics human neural development. Cell Stem Cell.

[110] Avaliani N., Sørensen A.T., Ledri M., Bengzon J., Koch P., Brüstle O., Deisseroth K., Andersson M., Kokaia M. (2014). Optogenetics reveal delayed afferent synaptogenesis on grafted human-induced pluripotent stem cell-derived neural progenitors. Stem Cells.

[111] Upadhya D., Hattiangady B., Shetty G.A., Zanirati G., Kodali M., Shetty A.K. (2016). Neural Stem Cell or Human Induced Pluripotent Stem Cell-Derived GABA-ergic Progenitor Cell Grafting in an Animal Model of Chronic Temporal Lobe Epilepsy. Curr. Protoc. Stem Cell Biol..

[112] Upadhya D., Hattiangady B., Castro O.W., Shuai B., Kodali M., Attaluri S., Bates A., Dong Y., Zhang S.C., Prockop D.J. (2019). Human induced pluripotent stem cell-derived MGE cell grafting after status epilepticus attenuates chronic epilepsy and comorbidities via synaptic integration. Proc. Natl. Acad. Sci. USA.

